# Association between *prodynorphin* gene polymorphisms and opioid dependence susceptibility: a meta-analysis

**DOI:** 10.1186/s12888-019-2272-7

**Published:** 2019-09-11

**Authors:** Chang-wang Wang, Min Ma, Wei-guang Lu, Ru-qin Luo

**Affiliations:** 1 Department of Psychiatry, Wuchang Hospital, South Luoshi Avenue 505#, Hongshan District, Wuhan, 430070 China; 2 Department of Anesthesiology, Zhongnan Hospital of Wuhan University, Wuhan, 430071 China

**Keywords:** *Prodynorphin*, Opioid dependence, Polymorphism, Meta-analysis

## Abstract

**Background:**

*Prodynorphin* (*PDYN*) gene polymorphisms have been linked with opioid dependence (OD) with conflicting outcomes, the aim of this study is to synthesize the existing evidence of the association between *PDYN* polymorphisms and OD susceptibility.

**Methods:**

Four databases including PubMed, EMBASE, Web of Science, and Wanfang were retrieved for relevant studies before August, 2018. All identified studies were evaluated using predetermined inclusion and exclusion criteria. Summary odds ratio (OR) and 95% confidence interval (95%CI) were calculated to appraise the association. Statistical analysis was performed using RevMan 5.3 software.

**Results:**

A total of seven case-control studies with 3129 cases and 3289 controls were recruited in the meta-analysis. For *rs910080*, *rs1997794*, *rs1022563*, and *rs2235749* polymorphisms of *PDYN* gene, there were six, four, five, and four studies eventually included, respectively. The findings indicated that *rs910080* polymorphism was significantly correlated with OD among Asian population under allelic model (A vs. G, OR = 1.30, 95% CI 1.04–1.62, *P* = 0.02, FDR = 0.05) and dominant model (AA+AG vs. GG, OR = 1.25, 95% CI 1.04–1.51, *P* = 0.02, FDR = 0.05). However, *rs1022563*, *rs1997794* and *rs2235749* polymorphisms did not appear to associate with OD susceptibility.

**Conclusions:**

There existed a significant association between *rs1022563* polymorphism and OD among Asian population. As the included studies were not adequate to guarantee a robust and convincing conclusion, future studies with larger sample size among more ethnicities are recommended.

## Background

Opioid dependence (OD), mainly characterized by persistent drug-taking and drug-seeking behavior, is a chronic brain disorder that has seriously negative social and health consequences [1]. A range of environmental factors like severe stressors, family separation, divorce, as well as death in the family have been considered to be associated with OD for a long time [2]. However, researchers observe that only a few individuals that were exposed to drugs will finally develop specific addictions [3]. Thus, there are some factors beyond environmental factors that can contribute to OD.

Actually, early in 1990s, the isolation of genes that encoded opioid peptide precursors had opened an era of molecular and genetic investigations of OD [4]. Family-based studies in different countries and regions revealed the heritability estimates for substance dependence of twins ranged from 30 to 70% [5]. Over the past years, numerous genes and single-nucleotide polymorphisms have been reported to be contributors of OD [6, 7].

Prodynorphin (PDYN), also known as preprodynorphin, is the precursor of neoendorphins and dynorphins [8]. The human *PDYN* gene is mapped to chromosome 20p13 [9]. *PDYN* gene knockout mice presented an increasingly explorative behavior in anxiety tests, which demonstrated the role of prodynorphin-derived peptides [10].

Since Zimprich et al. [11] initially reported the correlation between *PDYN* gene polymorphisms and OD, several researchers have subsequently replicated with inconsistent results [12–18]. The lack of convergent findings might ascribe to weak statistical power to ascertain a significant effect, population structure or between-study heterogeneity. Thus, this meta-analysis was conducted to provide a more precise estimation on the relationship of *PDYN* gene polymorphisms and OD.

## Methods

The procedures of the systematic review and meta-analysis were conducted in accordance with the Preferred Reporting Items for Systematic Reviews and Meta-Analyses (PRISMA) guidelines [19].

### Search strategy of literature

A systematic search of potentially relevant literature was carried out in PubMed, Web of Science, EMBASE, and Wanfang databases. The following search string was employed: (diamorphine OR opioids OR narcotic OR opium OR opiate OR heroin OR “Heroin”[Mesh]) AND (*PDYN* OR *prodynorphin*) AND (“Polymorphism, Single Nucleotide” [Mesh] OR SNP OR mutation OR polymorphism OR variant). No restriction on language was imposed. The search period was set from inception to August, 2018.

### Inclusion and exclusion criteria

Publications that were included in the present study had to satisfy the following criteria: (1) case-control studies looking into the association of *PDYN* gene polymorphisms and OD; (2) case participants with confirmed diagnosis of OD; (3) studies with sufficient genotype counts to estimate odds ratio (OR) as well as 95% confidence interval (95%CI). Accordingly, case-case studies, reviews, conference abstracts, as well as animal studies were excluded.

### Quality assessment

The quality of eligible studies was evaluated separately by two investigators (CW and MM) according to Newcastle-Ottawa Scale (NOS). A ‘star system’ was used to judge the quality of each study on the basis of selection, comparability, as well as exposure. The NOS ranges from zero to nine stars. Studies with five or more stars indicated a high quality.

### Data extraction

The following data were extracted independently by two investigators (CW and MM) from each qualified article: author, year of publication, country, ethnicity and gender of participants, sample size, and genotype distribution. Once encountering discrepancies, two authors re-checked the original articles until an agreement was achieved.

### Statistical analysis

Hardy-Weinberg Equilibrium (HWE) [20] of control subjects was calculated. The strength of association of PDYN gene polymorphisms and OD was presented as OR and 95%CI in allelic model (M vs. W), homozygous model (MM vs. WW), heterozygous model (MW vs. WW), dominant model (MM + MW vs. WW), as well as recessive model (MM vs. MW + WW). To evaluate the heterogeneity across included studies, Q-statistical test alongside I^2^ test were utilized. *P* < 0.1 and *I*^2^ > 50% suggested the heterogeneity was not significant, and the fixed-effect model could be applied, otherwise the random-effects model should be employed. Subgroup-analysis by ethnicity was performed to test if there was an ethnicity-specific effect. For multiple testing corrections, the Benjamini-Hochberg method was applied to control false discovery rate (FDR) [21]. Forest plots were made by RevMan 5.3 software. Funnel plots were exploited to assess the publication bias.

## Results

### Literature search

**Fig. 1:**
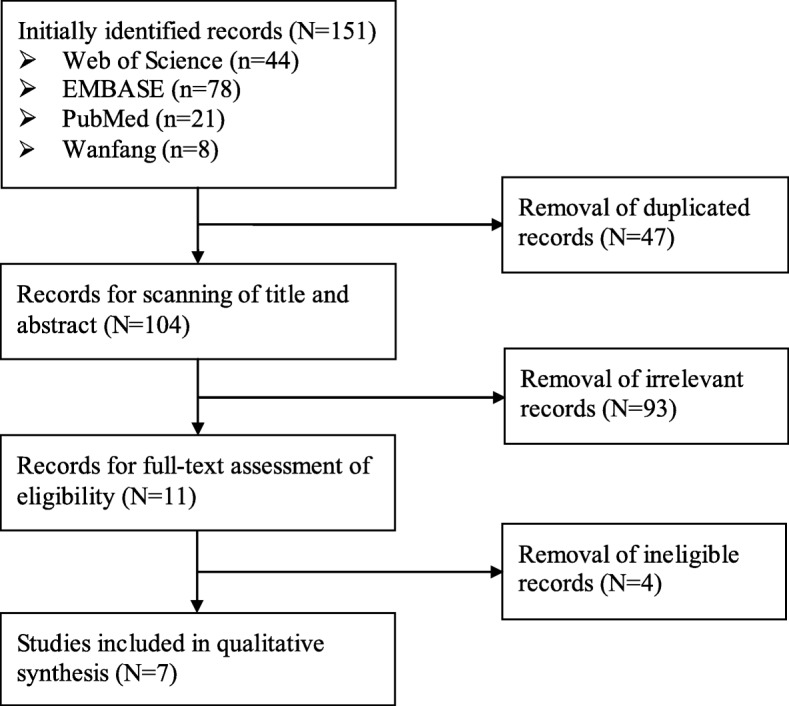
Flow chart of literature search and screen

An initial search of four databanks yielded 151 records, of which 44 from Web of Science, 78 from EMBASE, 21 from PubMed, and 8 from Wanfang. Forty-seven records were deleted because of duplication. Ninety-three irrelevant citations were removed from the remaining 104 records after title and abstract screening. Another 4 ineligible articles were erased after comprehensive assessment according to inclusion and exclusion criteria. Ultimately, 7 articles [12–18] were included in qualitative synthesis and meta-analysis. The process was displayed in Fig. 1.

### Main characteristics

The general characteristics of included studies were displayed in Table 1. A total of seven case-control studies [12–18] with 3129 cases and 3289 controls were included in the meta-analysis. Of the included studies, six [12–16, 18] were published in English and one [17] was in Chinese. It should be noted that Clarke et al.’s study [15] published in 2012 consisted of two different cohorts. The sample size of an individual cohort ranged from 119 to 1030 for cases, and 90 to 668 for controls. For *rs910080* polymorphism, Hashemi et al.’s study [14] was not in HWE. When it came to *rs1022563* polymorphism, three studies [16–18] were out of HWE. As to *rs2235749* polymorphism, three studies [14, 16, 17] were not in HWE. According to NOS, each study received five or more stars (Table 2).

**Table 1:** Characteristics of included studies

Study	Year	Country	Ethnicity	Gender	Sample size	OD (%)					Control (%)					HWE (*P*-value)
						M	W	MM	MW	WW	M	W	MM	MW	WW	
*Rs910080 (A/G)*																
Yuanyuan et al.	2018	China	Asian	Both	541/566	14.3	85.7	22.2	24.2	73.6	13.2	86.8	1.6	23.1	75.3	0.77
Nagaya et al.	2018	Malaysia	Asian	Male	459/543	16.1	83.9	34.9	25.3	71.2	12.4	87.6	2.4	20.1	77.5	0.07
Hashemi et al.	2018	Iran	Asian	Both	216/219	43.1	56.9	19.9	46.3	33.8	30.1	69.9	3.2	53.9	52.9	<0.01
Clarke et al. I	2012	USA	Caucasian	Both	1030/644	25.0	75.0	6.0	38.0	56.0	27.6	72.4	8.9	37.6	53.6	0.12
Clarke et al. II	2012	USA	African	Both	330/663	46.8	53.2	23.0	47.6	29.4	48.2	51.8	22.8	50.8	26.4	0.64
Wei et al.	2011	China	Asian	Both	304/301	21.9	78.1	4.9	33.9	61.2	15.3	84.7	1.3	27.9	70.8	0.18
Jia et al.	2010	China	Asian	Both	212/200	23.6	76.4	3.3	40.6	56.1	25.7	74.3	4.5	42.5	53.0	0.12
*Rs1997794(G/A)*																
Yuanyuan et al.	2018	China	Asian	Both	541/566	16.4	83.6	2.0	28.7	69.3	15.5	84.5	1.9	27.0	71.0	0.42
Nagaya et al.	2018	Malaysia	Asian	Male	459/543	48.2	51.8	22.7	51.2	23.1	50.9	49.1	26.0	49.9	24.1	0.97
Clarke et al. I	2012	USA	Caucasian	Both	1037/652	35.0	65.0	11.7	46.6	41.8	37.9	62.1	15.3	45.1	39.6	0.28
Clarke et al. II	2012	USA	African	Both	336/668	28.3	71.7	8.6	39.3	52.1	25.1	74.9	6.3	37.6	56.1	1.00
Clarke et al.	2009	China	Asian	Female	119/176	19.3	80.7	3.4	31.9	64.7	11.4	88.6	0.6	21.6	77.8	0.34
*Rs1022563(C/T)*																
Nagaya et al.	2018	Malaysia	Asian	Male	459/543	17.9	82.1	3.9	27.9	68.2	17.6	82.4	3.3	28.6	68.1	0.72
Clarke et al. I	2012	USA	Caucasian	Both	948/644	13.5	86.5	14.8	24.1	74.5	17.0	83.0	1.9	30.3	67.9	0.06
Clarke et al. II	2012	USA	African	Both	336/660	21.6	78.4	5.1	33.0	61.9	18.9	81.1	3.0	31.8	65.2	0.35
Wei et al.	2011	China	Asian	Both	304/300	16.3	83.7	3.9	24.7	71.4	17.0	83.0	0.7	32.7	66.7	<0.01
Jia et al.	2010	China	Asian	Both	212/90	19.1	80.9	4.7	28.8	66.5	42.8	57.2	6.7	72.1	21.1	<0.01
Clarke et al.	2009	China	Asian	Female	119/163	17.6	82.4	5.9	23.5	70.6	13.8	86.2	0	27.6	72.4	0.04
*Rs2235749(T/C)*																
Yuanyuan et al.	2018	China	Asian	Both	541/566	15.2	84.8	2.4	25.5	72.1	13.5	86.5	19.4	23.1	74.9	0.81
Hashemi et al.	2018	Iran	Asian	Both	216/219	38.2	61.8	7.9	60.6	31.5	37.2	62.8	4.1	66.2	29.7	<0.01
Wei et al.	2011	China	Asian	Both	304/300	21.9	78.1	5.3	33.2	61.5	15.3	84.7	1	28.7	70.3	0.07
Jia et al.	2010	China	Asian	Both	212/200	18.6	81.4	4.7	27.8	67.5	27.3	72.8	14	26.5	59.5	<0.01

*OD* opioid dependence, *M* mutant allele, *W* wild allele, *HWE* Hardy-Weinberg Equilibrium

**Table 2:** Quality of included studies

Item/Study	Nagaya et al. 2018 [13]	Hashemi et al. 2018 [14]	Clarke et al. 2012 [15]	Wei et al. 2011 [16]	Jia et al. 2010 [17]	Clarke et al. 2009 [18]	Yuanyuan et al. 2018 [12]
Adequate definition of cases	★	★	★	★	★	★	★
Representativeness of cases	☆	☆	☆	☆	☆	☆	☆
Selection of control subjects	☆	☆	☆	☆	☆	☆	☆
Definition of control subjects	★	★	★	★	★	★	★
Control for important factor or additional factor	★☆	★☆	★☆	★☆	☆☆	☆☆	★☆
Exposure assessment	★	★	★	★	★	★	★
Same method of ascertainment for all subjects	★	★	★	★	★	★	★
Non-response rate	★	★	★	★	★	★	★

★, a star given; ☆, a star not given

### Results of meta-analysis

The correlations of *PDYN* gene polymorphisms (*rs910080*, *rs1997794*, *rs1022563*, and *rs2235749*) and OD vulnerability were summarized in Table 3.

**Table 3:** Association between *prodynorphin* gene polymorphisms and opioid dependence

Genetic model	Association			FDR	Effect model	Heterogeneity	
	OR	95% CI	*P*-value			*I*^2^ (%)	*P*-value
*Rs910080*							
Overall							
A vs. G	1.16	0.94–1.41	0.16	0.28	R	81	<0.01
AA vs. GG	1.53	0.83–2.84	0.17	0.28	R	84	<0.01
AG vs. GG	1.06	0.95–1.19	0.32	0.32	F	32	0.18
AA + AG vs. GG	1.12	0.93–1.34	0.24	0.30	R	63	0.01
AA vs. AG + GG	1.52	0.85–2.70	0.16	0.28	R	83	<0.01
Asian							
A vs. G	1.30	1.04–1.62	0.02	0.05	R	70	0.01
AA vs. GG	2.21	0.96–5.09	0.06	0.07	R	76	<0.01
AG vs. GG	1.17	1.01–1.36	0.04	0.07	F	7	0.37
AA + AG vs. GG	1.25	1.04–1.51	0.02	0.05	F	40	0.15
AA vs. AG + GG	2.13	0.94–4.83	0.07	0.07	R	76	<0.01
*Rs1997794*							
Overall							
G vs. A	1.02	0.85–1.22	0.87	0.94	R	72	0.01
GG vs. AA	0.98	0.68–1.43	0.94	0.94	R	58	0.05
GA vs. AA	1.06	0.94–1.20	0.36	0.94	F	20	0.29
GG + GA vs. AA	1.07	0.89–1.30	0.46	0.94	R	55	0.06
GG vs. GA + AA	0.95	0.69–1.31	0.77	0.94	R	52	0.08
Asian							
G vs. A	1.06	0.76–1.46	0.74	0.74	R	78	0.01
GG vs. AA	0.90	0.65–1.23	0.50	0.63	F	49	0.14
GA vs. AA	1.10	0.91–1.32	0.34	0.63	R	51	0.13
GG + GA vs. AA	1.16	0.82–1.63	0.41	0.63	R	68	0.04
GG vs. GA + AA	0.89	0.68–1.17	0.41	0.63	F	39	0.20
*Rs1022563*							
Overall							
C vs. T	0.85	0.62–1.17	0.32	0.40	R	88	<0.01
CC vs. TT	1.29	0.57–2.89	0.54	0.54	R	75	<0.01
CT vs. TT	0.66	0.44–1.00	0.05	0.25	R	89	<0.01
CC + CT vs. TT	0.71	0.47–1.07	0.11	0.28	R	89	<0.01
CC vs. CT + TT	1.45	0.79–2.65	0.23	0.38	R	57	0.04
Asian							
C vs. T	0.80	0.47–1.37	0.42	0.50	R	91	<0.01
CC vs. TT	1.71	0.36–8.07	0.50	0.50	R	83	<0.01
CT vs. TT	0.54	0.26–1.13	0.10	0.35	R	92	<0.01
CC + CT vs. TT	0.60	0.29–1.25	0.17	0.35	R	93	<0.01
CC vs. CT + TT	2. 04	0.66–6.27	0.21	0.35	R	69	0.02
*Rs2235749*							
T vs. C	1.04	0.74–1.46	0.83	0.83	R	83	<0.01
TT vs. CC	1.34	0.42–4.25	0.62	0.83	R	85	<0.01
TC vs. CC	1.09	0.92–1.30	0.32	0.83	F	2	0.38
TT + TC vs. CC	1.05	0.78–1.41	0.75	0.83	R	64	0.02
TT vs. TC + CC	1.34	0.43–4.16	0.61	0.83	R	85	<0.01

*SNP* single nucleotide polymorphism, *OR* odds ratio, *CI* confidence interval, *F* fix-effect model, *R* random-effect model, *FDR* adjusted *P*-value for false discovery rate

Data synthesis for *rs910080* was available on six studies [12–17] with seven independent cohorts including 3092 cases and 3136 controls. Due to considerable heterogeneity, random-effect model was employed with the exception of heterozygous model. The pooled data indicated a null correlation between *rs910080* polymorphism and OD susceptibility under five genetic models. Subgroup analysis stratified by ethnicity suggested a significant association between *rs910080* polymorphism and OD across Asian population in allelic model (A vs. G, OR = 1.30, 95% CI 1.04–1.62, *P* = 0.02, FDR = 0.05) and dominant models (AA+AG vs. GG, OR = 1.25, 95% CI 1.04–1.51, *P* = 0.02, FDR = 0.05). While no correlation was detected under other models.

As for *rs1997794*, four studies [12, 13, 15, 18] with five cohorts involving 2942 cases and 2605 controls went into final data synthesis. Because heterogeneity between studies was significant, the random-effect model was applied. The result indicated that *rs1997794* polymorphism did not significantly associate with OD in five genetic models. Subgroup analysis stratified by ethnicity also yielded a null association.

In respect of *rs1022563*, five studies with six cohorts containing 2378 cases and 2400 controls satisfied the eventual data combination. Because of huge heterogeneity, random-effect model was utilized. *Rs1022563* polymorphism appeared to have no significant correlation with OD in five genetic models. Subgroup analysis by ethnicity as well indicated a non-significant association.

When it came to *rs2235749*, four studies [12, 14, 16, 17] with 1273 cases and 1285 controls carried out among Asian population were analyzed. OR and 95%CI were estimated by the random-effect approach due to significant heterogeneity. The pooled results indicated a null association between *rs2235749* polymorphism and OD susceptibility.

### Sensitivity analysis and publication bias

After studies that deviated from HWE were excluded, the corresponding OR did not reverse. Consequently, they were not removed from our meta-analysis. Sensitivity analysis confirmed the stability of the outcomes, where exclusion of individual dataset did not significantly change the overall effects. Furthermore, visual inspection of funnel plots did not identify substantial asymmetry for all the four loci under any genetic model.

## Discussion

OD affects the lives of millions of people worldwide and imposes a heavy burden on society [22]. Hence, seeking new strategies to prevent and treat OD is necessary. Investigation into the etiology of OD has focused on multiple areas. Genetic factors and environmental factors are both considered as contributors to OD. As a genetic factor, *PDYN* gene has received more and more attention [23].

Several studies across different populations have been performed on the association of *PDYN* gene polymorphisms and OD vulnerability, while it is quite regrettable that no convincing evidence is available. This inconsistency could be interpreted by several factors, including severity of diagnosis, limited sample size, inadequate power, ethnic heterogeneity, population stratification, as well as large phenotype range. The present study, with pooled data from Asian (Malaysia, Iran, and China), Caucasian (European American), and African (African American) populations, indicated that *rs910080* polymorphism was significantly correlated with OD among Asian population. However, *rs1022563, rs1997794* and *rs2235749* polymorphisms did not appear to associate with OD susceptibility.

*PDYN* gene codes ligand of opioid receptor. Functional variant in *PDYN* gene hampers the downstream signaling pathway and affects the overall biological networks, thus implicating in the pathophysiology of OD. Dynorphins are a cluster of posttranslational products of *PDYN* gene. They bind to all three types of opiate receptors, but exhibit considerable affinity to kappa opioid receptor [24]. Products of *PDYN* gene have been reported to inhibit neurotransmission and take part in mood regulation, stress responses [25], reward [26], and motor functions. Yuferov et al. [27] reported the mRNA of *PDYN* was rich in brain areas that were associated with drug-taking and drug-withdrawal. Wang et al. [28] found that acute intermittent morphine administration could raise *PDYN* and kappa opioid receptor mRNA level in the brains of rat models. Solecki et al. [29] observed that chronic exposure to heroin could lead to an increased *PDYN* mRNA in the nucleus accumbens shell and core of rats. All this evidence suggested that *PDYN* gene played a crucial role in the occurrence and development of OD.

Heterogeneity is a crucial issue when elucidating the outcomes of a meta-analysis. We attempted to minimize this issue by developing strict criteria for study inclusion, information extraction, as well as data analysis. Considerable heterogeneity between studies still existed despite subgroup analysis was stratified. Several potential reasons might give rise to the presence of heterogeneity. For demographics, age of individual study ranges greatly. For gender, although majority studies are mixed genders, some are focusing on only men or women. For genetic background, included studies are multi-national and many ethnic groups were represented within them. For environmental factor, participants from both developing countries and developed countries were incorporated into the present study.

### Study limitations

Although the current study has collected available data on the association between *PDYN* gene polymorphisms and OD predisposition, several limitations could not be neglected. First, the present outcomes were based on unadjusted estimates. Because raw data for confounding factors like age [30], gender [15], socioeconomic status [31] of the participants were unavailable, we could not perform the corresponding stratification analysis. Second, the number of participants of each included study was limited, thus lacking enough statistic power to guarantee the association. Third, we only investigated the role of four independent polymorphisms in *PDYN* gene. However, finding susceptibility polymorphisms and genes was a difficult task owing to the intricate mode of inheritance and multiple polymorphisms in various genes contributing to increased disease risk. OD, without exception, is a multifactor disorder determined by the synthetic effects and interactions of a series of polymorphisms, genes, and environmental factors. These interactions might conceal or enlarge the function of the included polymorphisms. Lastly, only seven articles from four databanks were searched for this study, potentially relevant studies published in other databanks might be missed.

## Conclusion

Taken together, the current study indicated the *rs910080* polymorphism was significantly correlated with OD among Asian population. However, *rs1022563*, *rs1997794* and *rs2235749* polymorphisms did not appear to associate with OD susceptibility. Due to limitations demonstrated above, the associations between *PDYN* gene polymorphisms and the risk of OD could not be entirely concluded. As the included studies were not adequate to guarantee a robust and convincing conclusion, studies with larger sample size among more populations are recommended.

## Data Availability

The datasets used and/or analyzed during the current study are available from the corresponding author Ru-qin Luo, ruqinluo@126.com.

## Abbreviations

CIs: Confidence interval
HWE: Hardy-Weinberg equilibrium
NOS: Newcastle-Ottawa Scale
OD: Opioid dependence
ORs: Odds ratios
*PDYN*: *prodynorphin*
